# At What Point in the Menstrual Cycle Are the Pelvic Floor Muscles at Their Weakest?

**DOI:** 10.3390/jfmk9030135

**Published:** 2024-08-08

**Authors:** Cristina Ojedo-Martín, Elena Sonsoles Rodríguez-López, María Barbaño Acevedo-Gómez, Edurne Úbeda-D’Ocasar, María Victoria de-Diego, Beatriz Lara

**Affiliations:** 1 Physiotherapy and Health Research Group (FYSA), Department of Physiotherapy, Faculty of Health Sciences-HM Hospitals, University Camilo José Cela, 28014 Madrid, Spain; cojedo@ucjc.edu (C.O.-M.); acevedogomez.maria@gmail.com (M.B.A.-G.); eubeda@ucjc.edu (E.Ú.-D.); 2 Department of Physiotherapy, Faculty of Health Sciences-HM Hospitals, University Camilo José Cela, 28014 Madrid, Spain; 3 Exeltis, 28108 Madrid, Spain; victoriadediego@hotmail.com; 4 Exercise Physiology Laboratory, Faculty of Health Sciences-HM Hospitals, University Camilo José Cela, 28014 Madrid, Spain; blara@ucjc.edu

**Keywords:** pelvic floor, strength, dynamometry, menstrual, hormones

## Abstract

Pelvic floor muscle (PFM) strength is a critical factor for optimal pelvic floor function. Fluctuations in strength values based on different phases of the menstrual cycle (MC) could signify a need for a paradigm shift in evaluating, approaching, and planning training. This research aims to examine and contrast the pelvic floor muscle strength during different phases of the menstrual cycle. A prospective observational study employing digital assessment with the modified Oxford scale and vaginal dynamometry measurements was performed, in order to assess the baseline strength and the contraction strength of the PFMs in eumenorrheic females at three different phases of the MC: the early follicular phase (EFP), the late follicular phase (LFP), and the mid-luteal phase (MLP). During two complete cycles, tympanic temperature and body weight were measured and the urinary luteinizing hormone concentration was tested to determine the time of ovulation. In total, 216 dynamometric measurements of PFM strength were obtained from eighteen nulliparous women (25.72 ± 5.03 years). There were no differences between the baseline strength (*p* = 0.886) and the contraction strength (*p* = 0.756) with the dynamometric speculum. In the post hoc analysis, the baseline strength, contraction strength, and strength showed no significant differences between MC phases. As no differences in PFM strength in women were found, the PFMs do not seem to be weaker at any time during the menstrual cycle. It appears that the assessment, establishment, and monitoring of a PFM training program could be initiated at any point in the cycle.

## 1. Introduction

Variations in female hormones throughout the menstrual cycle (MC) can impact various aspects of the cardiovascular, respiratory, thermoregulatory, and metabolic systems. Thus, they can also affect the physiology of the musculoskeletal system, due to the proven presence of estrogen and progesterone receptors in bones, skeletal muscles, ligaments, and the nervous system [1]. Research has suggested that estrogen can lead to a decline in muscular stiffness [2] and to increased muscle strength [3]. The MC is commonly divided into the follicular phase, with low levels of estrogen and progesterone; the ovulation phase, with high estrogen levels; and the luteal phase, characterized by high levels of both estrogen and progesterone [4].

Muscle performance during the MC has been evaluated [5,6,7]; however, the literature has not provided a robust conclusion regarding the impact of the MC on physical performance variations. There is no consensus on how the MC affects muscle flexibility and strength [8,9,10]. However, the decrease in estrogen levels—such as during the climacteric period—is linked to a weakening of the pelvic floor muscles (PFMs) and the possible development of urogynecological dysfunction such as urge incontinence [11]. Although it is challenging to distinguish the impact of decreasing estrogen levels during menopause from the overall aging process, there is evidence that the pelvic organs and their supportive muscular and connective tissues respond to estrogen [12].

The adverse effects of progesterone on female urinary tract function have been observed, as it is associated with heightened adrenergic tone leading to reduced tone in the ureters, urethra, and bladder. This may contribute to the exacerbation of urinary symptoms during the secretory phase of the MC. Additionally, progesterone might be linked to an increase in urgency during pregnancy; however, the exact mechanism remains unclear [13]. Sánchez et al. [14] have shown that the somatovisceral reflexes related to the bladder, urethra, and PFM are influenced by ovarian hormones, particularly estrogens. Their research emphasized the importance of understanding how estrogen impacts pubococcygeus muscle activity during urination. After menopause, women suffer a more rapid loss of muscle mass and, consequently, of strength. This fact could be explained, in part, by the decrease in satellite cells in skeletal muscle due to estrogen deficiency and its consequent decrease in estrogen receptor signaling [15,16]. However, the possible influence of progesterone on age-related skeletal muscle loss is unclear [17].

Pelvic floor training in women is essential to maintain good urogenital health and prevent dysfunctions such as urinary incontinence, fecal incontinence, organ prolapse, sexual dysfunction [18,19,20], or neurological problems such as pudendal neuralgia [21]. This set of muscles and tissues supports the pelvic organs, such as the bladder, uterus and rectum, and strengthening it can optimize PFM function. Pelvic floor dysfunction is a universal health problem with important implications for the family, economic, and social lives of those who suffer from them [22]. Individuals experiencing pelvic floor dysfunction confront multidimensional challenges that negatively affect diverse domains of their lives, thereby diminishing their overall quality of life. This encompasses notable impacts on sleep, physical function, and sexual activities [23]. These conditions affect between one in three to one in four women [24,25]. Understanding the potential influence of the hormonal cycle on PFM competence is crucial.

PFM assessment methods are diverse and minimally invasive, such as digital palpation, the use of translabial or intracavitary ultrasound, intravaginal clinical dynamometer, surface electromyography, or magnetic resonance imaging [26,27,28]. The International Continence Society does not currently consider any of the above methods to be the “gold standard” for measuring PFM strength [26]. Vaginal examination and force measurement are methods used to assess the strength of the PFMs [29,30,31]. Intravaginal palpation can be used to quantify pelvic floor muscle strength through checking the vaginal closing pressure. The modified Oxford scale (MOS) is a commonly employed technique to measure and evaluate pelvic floor muscle contraction and relaxation levels [32,33], which has good intra-observer reliability but its inter-observer reliability has been disputed [30]. Vaginal dynamometry assesses muscle strength using a speculum to measure the maximum force produced during PFM contraction [31,33] and appears to have strong intra-examiner [27] and inter-examiner agreement [32].

Fluctuations in strength values based on different phases of the cycle could signify a need for a paradigm shift in evaluating, approaching, and planning training regimens. Therefore, the objective of this study is to examine and contrast the pelvic floor muscle strength during different phases of the menstrual cycle in eumenorrheic women.

## 2. Materials and Methods

### 2.1. Study Design

A prospective observational study with an analytical approach was conducted, utilizing digital palpation and dynamometry techniques for data collection during three sessions at three different phases of the MC to assess PFM strength. The Ethics Committee of University Camilo José Cela (04_23_CISP, 12 April 2023; Madrid, Spain) approved this research. All women who started the study signed the informed consent form and obtained detailed information about the research planning.

### 2.2. Participants

To recruit participants, an advertisement with information about the study was published on the university campus of the Camilo José Cela University and on the university’s application for students and staff, such that they could contact us in case of interest. We subsequently conducted a phone interview with the women who expressed interest to verify their eligibility, followed by an in-person interview for documentation and material distribution. 

The inclusion criteria included women aged 18–35 years with regular menstrual cycles (self-reported length of 21–35 days [34,35] for each cycle for the past six months), nulliparous, with no history of taking oral contraceptives or any hormone-altering drug (including implants and patches) within the past six months, a body mass index (BMI) between 18 and 30, and a fat percentage between 18% and 35%. The study excluded pregnant women as well as those with pelvic organ prolapse, active or recurring genitourinary tract infections, urgent urinary incontinence, or polycystic ovary syndrome [36,37]; women experiencing psychological aversion, physical inability, or excessive vaginal muscle tension that precludes the insertion of a vaginal speculum [27,30,31]; those with a history of urogynecologic surgery, degenerative neurological disease, or any disease that could interfere with the measurement of the strength of the PFM; and those using analgesics or muscle relaxants. All participants attended three sessions for data collection.

### 2.3. Instrumentation and Data Collection

#### 2.3.1. Verification of Menstrual Cycle Phase

According to the hormonal fluctuations, three phases were recognized for measurements: early follicular phase (days 1–3 of the cycle, EFP), late follicular phase/ovulatory phase (days 13–14 of the cycle, LFP), and mid-luteal phase (day 21 of the cycle, MLP).

An initial gynecological assessment was carried out to rule out any of the exclusion criteria and to verify the MC phase with follicular scanning via transvaginal ultrasonography. Transvaginal ultrasound is the first choice for observing the ovary [38,39].

The participants used the calendar-based counting method to identify phases of the MC. This information was obtained using a mobile application (Mycalendar^®^, Period-tracker, Simple Design Ltd., Dewsbury, England) [40]. During the first contact with the participants, we introduced them to the application and asked them to record the dates of their last cycles. The duration of the MC was recorded for a minimum of four months for valid characterization. This was possible, as the measurements began at least four months after the first interview. Basal body temperature (BBT) and body mass changes were measured and recorded every morning immediately after waking up, using a digital tympanic thermometer (B08CH7BF7Y) and a digital scale (B0786KCWFJ). Participants collected these data for two complete MCs. Finally, measurement of the luteinizing hormone (LH) surge in urine was recorded. The women in this study used an ovulation predictor kit (B0872BL6FG) and their urine was collected at the same time of day (mid-morning) from day 9 of the MC until a positive test result occurred. The test results were sent by photo to the investigator, in order to avoid misinterpretation. In most cases, ovulation has been shown to occur within 14–26 h of the urinary LH peak [41]. Ultrasonographic measurements of ovarian volume, antral follicle, and endometrium thickness were collected to verify the MC phase with ultrasound (Samsung^®^ HS40, Suwon, Republic of Korea).

All these methods helped to align the participants’ cycles; therefore, despite different cycle lengths, participants performed testing in the same cycle phases. The order of the first MC phase was randomized for each participant and counterbalanced (five started in the EFP, seven started in the LFP, and six started in the MLP). Simple randomization was performed using computer-generated random numbers (randomizer.org), and each participant was scheduled for her first measurement based on the phase of the cycle in which she was assigned. 

#### 2.3.2. Sociodemographic Data and Questionnaires

On the day that the participants were scheduled to receive the material, book the scheduled appointments, and be evaluated by the gynecologist, they were measured using a body composition analyzer (Tanita BF 350, Tanita Corporation, Tokio, Japan), where their weight and fat percentage were recorded. 

Additionally, the women engaged in an online questionnaire distributed through the Survey Monkey^®^ platform (San Mateo, CA, USA), with the aim of collecting anthropometric and sociodemographic data, their medical history (common diseases, urinary tract infections, gynecological and obstetric history), various information on their menstrual cycles (duration, days of menstruation, age, weight, height and BMI) and physical activity through the International Physical Activity Questionnaire (IPAQ) [42]. 

#### 2.3.3. First PFM Assessment: The Modified Oxford Scale (MOS)

The participants, with empty bladders, were positioned in a supine orientation on a stretcher. Pillows were placed under the head and hips, and the knees were gently flexed and supported by a roller. The lumbar spine was maintained in a neutral alignment. A bidigital examination involving the use of a lubricant was conducted to evaluate the global voluntary contractility of the pelvic floor muscles by an expert physiotherapist, who was unaware of the participant’s cycle phase.

Women received instruction and guidance on the proper technique for performing a voluntary contraction of the pelvic floor muscles, including education on pelvic floor anatomy and function, using models or drawings if necessary. The closing pressure at the vaginal level and the contractile capacity of the PFMs were also verified by means of visual inspection and internal palpation, for which the subject was asked to pull the PFMs inward and upward with as much force as possible. In situations where the participant did not exhibit the appropriate muscle contraction, they were instructed to perform it correctly using biofeedback, avoiding any parasitic contractions or pelvic displacement.

MOS grades pelvic floor strength into 6 levels, from 0, referring to no contraction, to 5, which is an elevation of the PFMs against the manual resistance of the physiotherapist. [43,44]. 

#### 2.3.4. Second PFM Assessment: Intravaginal Clinical Dynamometry

The strength of the PFM was assessed using a clinical dynamometer constructed from acrylonitrile butadiene styrene and polycarbonate materials (Pelvibex^®^, Hernani, Spain, P201130449), comprising an adjustable vaginal speculum (8.5 cm long, 2 cm wide, 26 mm diameter and 12 g weight of speculum branches), where an inductive displacement sensor was connected to a spring of established rigidity [31]. The speculum was introduced into the vaginal cavity respecting its anatomical orientation and in its closed position and, once the two branches were introduced, they were allowed to open. The two branches opened at an angle [27]. The force exerted by the women was measured in Newtons (N). The dynamometer demonstrated high intrarater reliability of 0.942 and interrater reliability of 0.937. This intravaginal clinical dynamometer used to quantify pelvic floor muscle strength exhibits good reliability and validity [31].

The speculum was for individual use, and prior to evaluation, the speculum branches were properly disinfected (Steranius 2%) and covered with a speculum-specific sheath (two-finger gloves of opaque polyethylene for gynecological examination, Legueu^®^ type) and water-soluble gel (Sulky^®^, Kennesaw, GA, USA). Two consecutive measurements of PFM contraction force were recorded using the dynamometric speculum. The rest period between the two measurements was 30 s. Two values were recorded for each measurement: the baseline value of the passive force exerted by the PFM after 5 s of opening the two blades of the speculum in the vaginal canal (baseline strength) and the maximal voluntary contraction recorded by the device for 10 s (contraction strength) [27,31,43]. The instruction given was “squeeze the pelvic floor muscles as hard as you can” [45]. The strength of PFM contraction was measured as the difference between the maximal voluntary contraction strength and the baseline resting strength. The three variables, following the definition of Romero-Cullerés [27], are represented as baseline strength, contraction strength, and strength. All measurements were performed by a single evaluator—namely, a physiotherapist specializing in pelvic floor rehabilitation with more than 10 years of experience—who was unaware of the participant’s cycle phase.

### 2.4. Data Analysis

The G*power software version 3.1.9.6 (Kiel University, Kiel, Germany) was used to calculate the sample size. Then, a two-tailed hypothesis with an effect size of 0.80, an α error probability of 0.01, and statistical power of 0.99 were employed for the sample size calculation. According to these parameters, eighteen participants were necessary to complete this study. The sample size used in previous similar research was also considered [43,44].

The researchers employed IBM Statistics Package for Social Science, v.26 (IBM Corp, New York, NY, USA). The data are described using mean and standard deviation, with a 95% confidence interval (95% CI). The normality was tested using Shapiro–Wilk tests. The Friedman test was used to compare the dynamometric values across different menstrual phases. To further investigate differences between specific time points, the Games–Howell post hoc procedure was employed. Additionally, Pearson’s correlation coefficient was calculated to assess the relationships among the quantitative variables. The confidence level was set at 95%, and the threshold for statistical significance was *p* < 0.05.

## 3. Results

A total of twenty-two women were recruited, four of whom were excluded from the study. One of them was due to vaginismus and an inability to insert the dynamometric speculum, one due to a tight hymenal ring, and two did not complete all three measurements. All participants were nulliparous and seventeen of them were nulligravidae. The final sample comprised 18 nulliparous females with a mean age of 25.72 (5.03) years, body mass of 60.81 (11.57) kilograms, body height of 1.65 (0.07) meters, a body mass index (BMI) of 22.13 (3.26) kg/m^2^, and a body fat percentage of 26.85 (6.30) percent. The average length of the MC was 28.61 (1.03) days, with a range of 28–31 days, and the mean duration of menstruation was 4.88 (1.07) days, with a range of 3–7. The mean age at menarche was 12.55 (1.61) years, with a range of 10–16 years. Two women presented with constipation and three experienced stress UI, and none had undergone gynecological surgery. Five women had suffered from a urinary tract infection in the past, but none suffered from recurrent urinary tract infections. Ten reported experiencing painful menstruation, and eleven detailed experiencing premenstrual syndrome (PMS). Among them, two were professional sportswomen, two had been professional sportswomen in the past, and twelve engaged in regular physical activity. The level of physical activity performed by the participants, according to the self-reported IPAQ, indicated that seven women performed a high level, seven of them a moderate level, and four a low level of physical activity. Eight of them reported playing sports such as soccer, swimming, volleyball, and athletics. All participants were white people. A total of 216 dynamometric measurements were carried out in the different phases of the MC.

**Figure 1:**
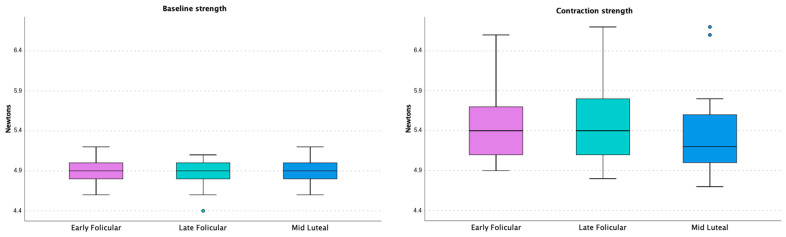
Representation of PFM strength according to menstrual cycle phase. The graph shows a box plot with measures of baseline strength (**left**) and contraction strength (**right**) in Newtons.

According to the MC phase, Table 1 shows the PFM strength measured with the dynamometric speculum and the score on the modified Oxford Grading Scale for each phase. Similar values were obtained in the first and second measurements (*p* > 0.05). The analysis was not significant (*p* > 0.05), indicating that there were no differences between the initial measurement and the contraction force with the dynamometric speculum. In the post hoc analysis, the baseline strength, contraction strength, and strength did not significantly differ between MC phases (Figure 1). There were no significant differences in measurements according to stress UI, constipation, menarche, painful menstruation, PMS, or level of physical activity.

Significant direct associations in the three phases of the MC were found between the maximum voluntary strength, the strength between measurements, and the score on the MOS (*p* < 0.05). During the EFP, younger women seemed to have more PFM strength (r = −0.558; *p* = 0.016); furthermore, the baseline strength seemed to be lower when women had a higher weight (r = −0.713; *p* = 0.001) and a higher BMI (r = −0.604; *p* = 0.008).

## 4. Discussion

This study sought to assess the influence of hormonal fluctuations on PFM strength measured by intravaginal dynamometer. The results suggest that the MC does not seem to modify the passive component and the strength of the PFM.

In terms of the assessment conducted, there were no notable disparities observed between the initial and subsequent measurements. This could imply that an adequate amount of time was allowed for rest in between these assessments. Our procedure involved a 30 s interval of rest between the two measurements.

Previous research yielded comparable findings when employing rest periods of 30 s [27], 1 min [46], 2 min [47,48], and 3 min [32]. The consistent and reliable measurements indicate that dynamometers are a dependable means of evaluating PFM function [49].

In line with previous studies [46,50], no statistically significant differences were found in the phases of the MC. Dos Reis et al. [46] obtained similar results when comparing the three measurements between 7-day intervals, although the study was based on the reliability of dynamometric variables. The only variable in which they obtained significant differences was the impulse of contraction of the PFM. This variable could not be used in our study, as we used a different dynamometer. Micussi et al. [50] evaluated the muscle tone and maximum voluntary contraction of PFMs using surface electromyography. The maximum voluntary contraction was not different across phases, but muscle tone was significantly lower during the follicular and ovulatory phases than the luteal phase. Our findings did not indicate these differences, although it is important to highlight that electromyography assesses the electrical activity of muscle membranes stimulated by neuromuscular activation, thus representing a more reliable measurement tool.

Before the measurements, participants in our study were given directions to become acquainted with the tasks but were specifically instructed not to engage in pelvic floor exercises during this time. This was done to ensure that any potential changes in active strength could not be attributed to learning effects.

The baseline strength measured in our study did not change during the phases. The passive force exerted by the walls of the vagina is due to its tubular anatomical arrangement and to the fact that it is supported by the PFMs and connective tissue, such that its viscoelastic component varies between subjects. The clinical dynamometer used had two branches that opened at an angle and produced reliable measurements of passive PFM properties [51]. There were no differences between the first and second measurements of baseline strength and contraction strength. Czyrnyj et al. [52] reported that task familiarization did not significantly influence the active or passive properties of the pelvic floor muscles, as measured through intravaginal dynamometry, in a sample of nulliparous women, as compared to the findings in this study. 

In our results, younger women seemed to have more PFM strength during the EFP. Although there are no studies correlating age between the phases of the MC, Quartly E. et al. [53] described the maximum strength and endurance in PFM of continent women, and they found no correlation between maximum strength of the PFM and age, although they did find greater endurance in the PFM of women older than 40 years, relating this fact to the increase in the proportion of type I fibers in the levator ani muscle. Another study [54] that analyzed the functional changes of PFM in different reproductive states of women only found a significantly lower basal PFM tone in multiparous women and in women over 46 years old. In addition, we found that baseline strength appeared to be lower when women had higher weight and BMI, also during the EFP. This finding could be related to the existing evidence that higher body weight increases the risk of urinary incontinence [55], but our sample size is too small to obtain conclusive data. 

The role of estrogen in the metabolism of collagen influences changes in the concentration and quality of collagen, as well as the morphology of connective tissue [56]. The main component of connective tissue is collagen, more specifically focusing on the fascia and ligaments of the pelvic region [57]. From this perspective, passive force (baseline strength) could have been influenced by hormonal changes during the MC, but no changes were found. Vita et al. [58] examined the influence of female hormones on the thickness and elasticity of the fasciae in users and non-users of hormonal contraceptives and found a borderline significant difference between the first (during the first 5 days of the MC) and second (between day 9 and day 13) measurements of the fascia lata among the non-users, and no significant changes in the thicknesses of the thoracolumbar and plantar fasciae. 

Other research has produced comparable findings when examining the impact of the MC on different muscle groups. Piasecky et al. [59] conducted a study to investigate potential changes in neuromuscular function and performance of the vastus lateralis motor unit using intramuscular electromyography across the MC in women with regular periods, revealing that there were no significant alterations observed in knee extensor muscle strength, power, and force control. Another research [60] examined the diversity in vertical jump measurements and running distance but found no discernible distinctions. Pelvic floor dysfunction in neurological conditions can worsen neurological issues associated with bladder and bowel control, and precise evaluation of these disorders may improve the effectiveness of rehabilitation interventions [61].

Recent systematic reviews and meta-analyses have emphasized the absence of consistent high-quality research, as well as a largely negligible difference in strength throughout the eumenorrheic cycle [5,6,62].

Concerning the clinical application of these results, the absence of changes in baseline strength and contraction strength recorded in the different phases indicates that it could be possible to perform a clinical assessment at any time during the female cycle, without doubting a possible hormonal influence.

### Strengths and Limitation

To the best of our knowledge, this is the first study to report adaptations of PFM strength using an intravaginal dynamometer across three timepoints of the MC, including monitoring and detection of the luteinizing hormone surge associated with ovulation. However, the measurement of serum estrogen and progesterone concentrations was not conducted during the luteal phase. This test is essential for confirming a regular ovulatory cycle, in accordance with recommended criteria for experimental procedures across the MC [63]. It would also be interesting to carry out measurements in women in the climatic or menopause stages.

A relevant fact is that the measurements in this study were performed only in the supine decubitus position, without performing any movement or using any other posture. Therefore, the results may change if the strength were evaluated during any activity or posture. To mitigate potential learning effects, the menstrual cycle phase during which participants commenced their measurements was randomized.

The sample consisted of physically active white women, which may make it difficult to generalize the results, thus reducing their external validity. PMS and dysmenorrhea were self-reported; validated questionnaires should be used to confirm the presence of PMS and dysmenorrhea in future research. 

## 5. Conclusions

Pelvic floor muscle strength in women seem to remain consistent across the different phases of the menstrual cycle and between measurements. Although hormonal influence may determine changes in the active or passive PFM properties during the menstrual cycle, the PFMs do not seem to be weaker at any time during the menstrual cycle, according to our results. It appears that the clinical assessment, establishment, and monitoring of a PFM training program could be initiated at any point in the cycle. 

To the best of our knowledge, this is the first time that a dynamometric study has been performed during the different phases of the menstrual cycle, and health professionals should conduct clinical studies in which pelvic floor strength is studied during different activities to continue research on this important factor for women. 

## Figures and Tables

**Table 1:** PFM strength measured with the modified Oxford Grading Scale and dynamometric speculum, according to the menstrual cycle phase.

	EFP			LFP			MLP			
	Mean (SD)	95% CI	*p* Value ^a^	Mean (SD)	95% CI	*p* Value ^a^	Mean (SD)	95% CI	*p* Value ^a^	Between Phases *p* Value ^b^
Strength (Modified Oxford Grading Scale)	3.67 (0.49)	(3.43–3.91)	-	3.67 (0.59)	(3.37–3.96)	-	3.56 (0.62)	(3.25–3.86)	-	0.449
Baseline strength (N) 1st measurement *	4.84 (0.10)	(4.79–4.89)	0.333	4.91 (0.18)	(4.81–5.00)	0.790	4.83 (0.12)	(4.77–4.89)	0.248	0.513
Baseline strength (N) 2nd measurement *	4.91 (0.15)	(4.84–4.99)		4.89 (0.18)	(4.80–4.98)		4.87 (0.16)	(4.79–4.95)		0.886
Contraction strength (N) 1st measurement *	5.25 (0.30)	(5.10–5.40)	0.230	5.44 (0.68)	(5.11–5.78)	0.381	5.42 (0.74)	(5.05–5.78)	0.321	0.407
Contraction strength (N) 2nd measurement *	5.46 (0.47)	(5.22–5.69)		5.52 (0.54)	(5.25–5.79)		5.47 (0.66)	(5.14–5.80)		0.756
Strength (N) 1st measurement *	0.41 (0.29)	(0.27–0.55)	0.327	0.54 (0.69)	(0.20–0.89)	0.414	0.58 (0.79)	(0.19–0.98)	0.775	0.727
Strength (N) 2nd measurement *	0.54 (0.53)	(0.28–0.81)		0.63 (0.59)	(0.33–0.92)		0.60 (0.74)	(0.23–0.97)		0.717

CI, Confidence interval; EFP, Early follicular phase; LFP, Late follicular phase; MLP, Mid luteal phase; N, Newtons; SD, Standard deviation. * PFM strength measured with the dynamometric speculum. Data from continuous variables are presented as the mean (standard deviation). Significance level was set at *p* < 0.05. (a) Difference between 1st and 2nd measurement according to the *p* value based on the Games–Howell post hoc test. (b) Difference between phases according to the *p* value based on Friedman test.

## Data Availability

The data presented in this study are available upon request from the corresponding author. The data are not publicly available due to privacy and ethical restrictions.
